# Recent Advances in Genetic Testing and Clinical Management of Hereditary Breast and Ovarian Cancer (HBOC) in India

**DOI:** 10.1002/cam4.71497

**Published:** 2026-07-24

**Authors:** Prathika Sherigar, Srikrishna Kedlaya Herga, Ananth Pai, Sharada Mailankody, Karthik S. Udupa

**Affiliations:** ^1^ Department of Medical Oncology, Manipal Comprehensive Cancer Care Centre, Kasturba Medical College (KMC) Manipal Academy of Higher Education Manipal Karnataka India; ^2^ Department of Public Health Genomics, Manipal School of Life Sciences Manipal Academy of Higher Education Manipal Karnataka India

**Keywords:** *BRCA*, genetic counseling, genetic testing, hereditary breast and ovarian cancer, next‐generation sequencing

## Abstract

**Background:**

Hereditary breast and ovarian cancer (HBOC) syndromes, responsible for 5%–10% of all breast and ovarian cancers in the general population, are largely associated with pathogenic variants of the BRCA1 and *BRCA2* genes. Yet, the role of other cancer susceptibility genes highlights the genetic etiology of HBOC as complex, thus requiring thorough investigation beyond these main mutations, highlighting a need for a comprehensive genetic assessment in disease management strategies.

**Summary:**

In India, advances in genetic research and clinical management have significantly impacted the knowledge of HBOC. A definitive prevalence of BRCA1*/2* mutations among Indian populations has catalyzed the adoption of genetic counseling for precision diagnosis and treatment strategies in recent times, with collateral support extended from communities among oncologists, geneticists, and reproductive medicine specialists. The integration of next‐generation sequencing and multiplex gene panels creates a platform for identifying high‐risk subjects, leading to individualized care pathways and enhanced disease management plans. These programs have increased access to essential services such as genetic counseling, multidisciplinary management, and fertility preservation to provide holistic care to HBOC patients.

**Key Message:**

Future efforts should explore further the genetic heterogeneity of HBOC in Indian populations. There needs to be wider access to genetic testing and counseling services, and the implementation of strong, ethical policy guidelines for equitable use of genetic information. Through the creation of innovative, collaborative methods, these measures have tremendous potential to improve patient care, early detection, and outcomes for individuals affected by HBOC in India.

## Graphical abstract

A multidisciplinary approach to hereditary breast and ovarian cancer (HBOC) involving genetic testing, counseling, and targeted therapy enables effective risk reduction and personalized disease management.

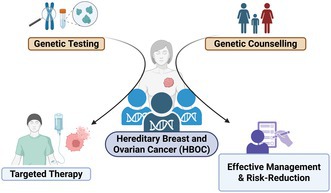

## 1 Introduction

Gynaecologic cancers impose significant social and economic impacts, especially in low‐ and middle‐income nations where late‐stage diagnosis is prevalent and access to care is restricted [1]. Ovarian cancer, usually asymptomatic until late stages, continues to be one of the deadliest gynecologic cancers, disproportionately affecting reproductive health, quality of life, and family dynamics. Breast cancer, although not a gynecologic cancer, is the most commonly diagnosed cancer among women worldwide and contributes substantially to cancer‐related morbidity and mortality. Globally, ovarian cancer caused around 295,000 new cases and nearly 185,000 deaths in 2018 [2]. Breast cancer, together with ovarian cancer, was another major contributor to cancer morbidity, accounting for more than 2 million new cases in 2018, accounting for 11.6% of all new cancer diagnoses across genders, as per the WHO. While most of these cancers arise sporadically, inherited genetic predisposition underlies approximately 5%–10% of breast and up to 15% of ovarian cancers [3]. Research into hereditary breast and ovarian cancer syndrome (HBOC) not only focuses on identifying individuals predisposed to cancer but also encompasses aspects such as optimizing current treatment strategies for breast cancer, implementing preventive measures against future cancers, conducting risk assessments, and establishing protective measures for family members at risk [4].

**FIGURE 1:**
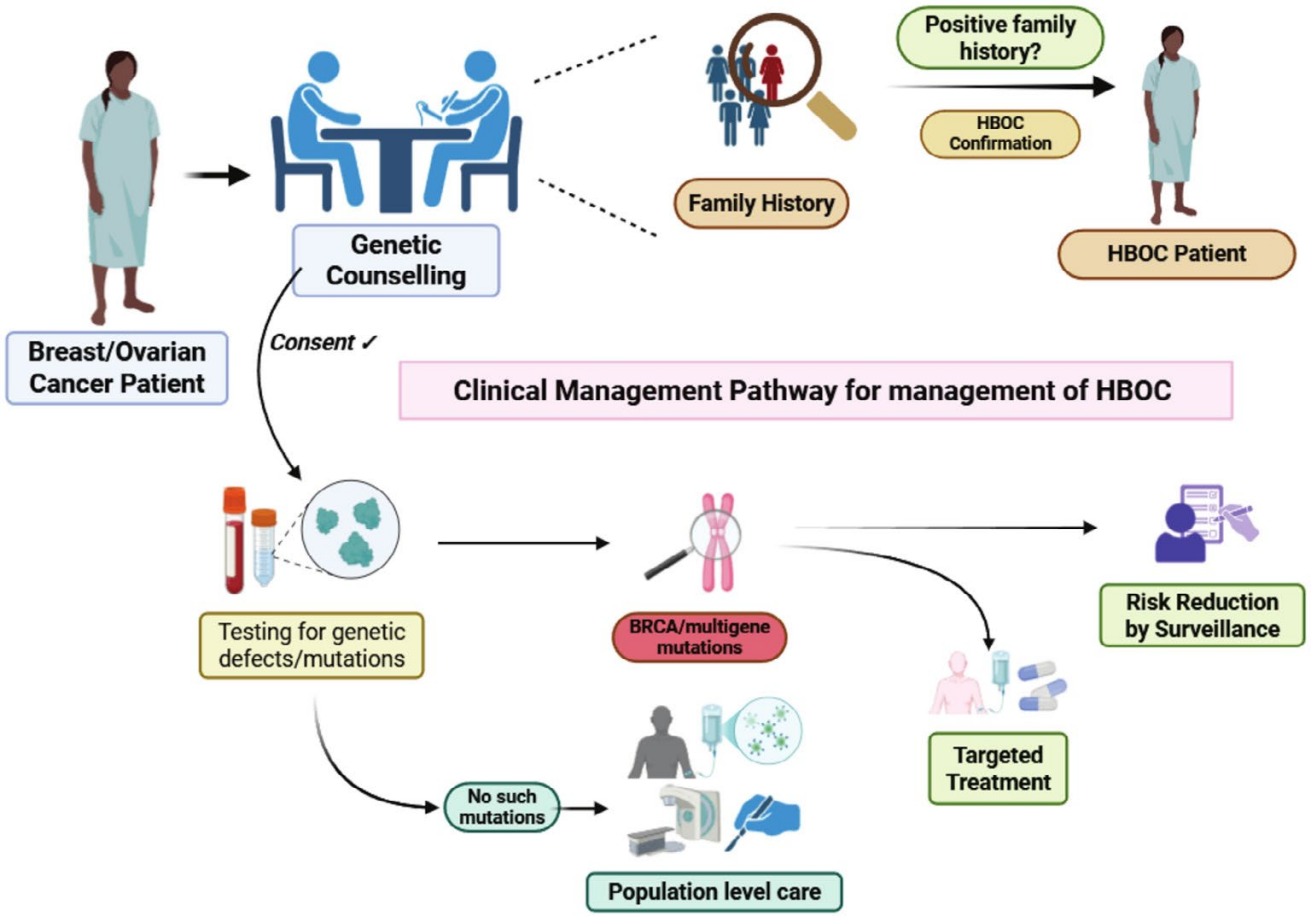
Clinical management flowchart for hereditary breast and ovarian cancer (HBOC). Subjects with breast or ovarian cancer are referred for genetic counseling to assess their risk for a hereditary syndrome. Counseling involves obtaining consent for genetic testing and assessing family history for associated cancers. If a pathogenic BRCA1/2 or multigene mutation is detected after testing and HBOC Confirmation is made, patients are considered HBOC patients and may undergo risk‐reducing interventions or targeted therapies. Mutation‐negative individuals follow general population screening protocols.

HBOC is an inherited condition attributed to elevated breast and ovarian cancer risk, accounting for a significant portion of these cancer types. The most extensively studied genes associated with HBOC are BRCA1 and *BRCA2*, which carry a risk for mutation carriers [3, 5]. Hereditary susceptibility genes also contribute to male breast cancer risk, although penetrance varies across genes [6]. Advancements in high‐throughput sequencing have transformed diagnostic methods. Many laboratories now regularly conduct multigene panel testing to diagnose HBOC. The French Genetic and Cancer Group has outlined guidelines for detecting and preventing hereditary cancers, recommending a panel of 13 genes for HBOC diagnosis [7], including BRCA1, *MSH2*, *BRCA2*, *PMS2*, *RAD51C*, *TP53*, *CDH1*, *PTEN*, *RAD51D*, *PALB2*, *MLH1*, *MSH6*, and *EPCAM* [8, 9]. Pathogenic or likely pathogenic variants in genes such as *TP53* and *PTEN* have also been implicated in familial breast cancers [10]. A considerable proportion of familial breast cancer remains unexplained by *BRCA1/2* mutations, suggesting the involvement of additional susceptibility genes [11]. Additionally, recent research has indicated the pathogenicity of mutations in *BRIP1*, *RAD51C*, *and RAD51D* in ovarian cancer patients, collectively accounting for approximately 2% of these cases [12]. These links highlight the importance of incorporating the genetic testing workflow in diagnosing and managing gynecological cancer cases suspected of having a hereditary gene mutation (Figure 1). Advancements in genetic testing for hereditary cancer predisposition genes have significantly impacted cancer treatment by enabling the detection of these conditions. This has facilitated the development of optimized cancer screening and prevention methodologies [13]. Next‐generation sequencing (NGS), which employs clonal amplification coupled with massive parallel sequencing, has emerged as a widely used technique. It enables the study of multiple genes at a reduced cost and with a shorter or more efficient turnaround time, simplifying the testing process for gene mutations in both germline and somatic cells [14].

Global advances in HBOC management have led to the development of multiple international clinical guidelines that provide recommendations on genetic counseling, testing, surveillance, risk‐reducing surgery, and systemic therapy [15, 16, 17]. In parallel, multigene panel testing studies have further refined the identification of hereditary cancer susceptibility variants [13]. According to GLOBOCAN 2020 estimates, breast and ovarian cancers continue to contribute substantially to the global cancer burden, highlighting the importance of effective genetic risk assessment, early detection, and preventive strategies [18]. However, these guidance documents and testing approaches may not be directly applicable to all populations due to differences in genetic architecture, healthcare infrastructure, and access to genetic services. Additionally, studies analyzing diagnostic models, genetic counseling, testing, and sequencing approaches for mutation detection within Indian populations are also scarce. There have been notable advancements in diagnostic testing, encompassing new treatments, testing methodologies, and evolving insights into genetic associations. In response to these developments, a set of best practice guidelines has been established to help clinical laboratories navigate issues associated with HBOC testing. The 32 published international guidelines offer recommendations for genetic counseling, risk reduction methods, and management specific to BRCA‐mutated breast and ovarian cancers. While BRCA mutations are also associated with other malignancies such as prostate and pancreatic cancers, we deliberately focus on the context of *BRCA* mutations in HBOC. A brief mention of these broader cancer risks is included for completeness, but the primary emphasis remains on breast and ovarian cancers due to their higher clinical and public‐health relevance in India. However, they may not fully cater to the unique challenges and contexts present in India. Consequently, there is a need to adapt and tailor these recommendations to the Indian healthcare landscape. India‐specific expert consensus and position statements highlight several gaps in the current delivery of HBOC care, including (1) a scarcity of structured, accredited genetic‐counseling programmes and consequently limited access to trained genetic counselors for pre‐ and post‐test counseling [17]; (2) concentration of clinical genetic testing and counseling services within urban tertiary cancer centres, leaving patients outside these hubs with restrcted access [19]; (3) wide heterogeneity in result‐delivery pathways and restricted patient access to recommended surveillance and risk‐reducing interventions (breast MRI, prophylactic mastectomy, salpingo‐oophorectomy, chemoprevention) because of infrastructure, cost and sociocultural barriers [20]; and (4) substantial opportunities to improve care by expanding cost‐effective multigene testing, developing a trained genetic‐counseling workforce, integrating HBOC services into national cancer control efforts, and increasing awareness among patients and providers [21]. By leveraging recent advancements in HBOC management, efforts are being made to evaluate current testing practices and referral workflows, propose effective testing methodologies, and facilitate the testing of BRCA mutations in India. The overarching objective is to enhance treatment outcomes and improve patient care by refining HBOC testing approaches within the Indian healthcare setting [22]. This comprehensive approach to HBOC research highlights the multifaceted nature of addressing genetic predisposition to cancer, as well as its applications in patient care and familial risk management [23].

This study aims to review recent advancements in genetic testing and clinical management specific to HBOC within the Indian context. This involves evaluating developments in genetic testing techniques, counseling practices, and clinical management strategies tailored to the Indian population. By synthesizing available research and clinical data, this study aims to provide insights into the current state of HBOC management in India, identify knowledge gaps or areas for improvement in practice, and suggest potential avenues for further research or enhancements in clinical care.

## 2 Genetic Landscape and Epidemiology of HBOC

HBOC is attributed to an inherited predisposition to elevated risks of breast cancer, especially before the age of 50 [24, 25]. The primary genetic underpinnings of HBOC involve pathogenic variants in two key genes, known as BRCA1 and *BRCA2*. *BRCA* is known as *BR*east *CA*ncer gene. BRCA1, located on chromosome 17q21.3, was identified in 1994 [26], while *BRCA2*, located on chromosomes 13q12‐13, was discovered in 1995. BRCA1 resides on chromosome 17q21, positioned close to the centromere on the long arm of the chromosome. It comprises 22 exons. The protein, comprised of 1863 amino acids encompasses a central N‐terminal RING finger domain, a C‐terminal BRCT domain, and includes exons 11–13 [27]. Similarly, *BRCA2* comprises 27 exons. It codes for a protein consisting of 3418 amino acids. The N‐terminal region of *BRCA2* has a transcription activation domain, whereas the substantial portion, encoded by exon 11, has eight conserved motifs referred to as BRC repeats that interact with *RAD51* [28].

Both genes are classified as tumor suppressor genes, playing crucial roles in halting inappropriate cell growth and division. Actionable variants in both genes significantly raise the chance of breast and ovarian cancers (BOC) in females. However, it is also of concern to men, who might face increased risks of breast and prostate cancer with pathogenic variants in this gene. Furthermore, individuals with BRCA1 or *BRCA2* mutations may experience a slight elevation in the risk for different cancers [29]. These pivotal DNA repair genes follow a monogenic inheritance pattern, meaning mutated alleles are passed on to 50% of descendants, with the likelihood of mutation influenced by factors such as familial disease frequency, age of disease onset, and affected organs, such as the breast and ovary [23].

Not all families with a history of multiple BOCs carry mutations in BRCA1 and *BRCA2*. For instance, individuals inheriting a germline mutation in BRCA1 or *BRCA2* may not develop cancer because they have not acquired the additional mutation required to disrupt the function of the gene and trigger tumor formation. The inheritance pattern of cancer within families can skip generations when such mutations are present. Nevertheless, individuals carrying these mutations, regardless of whether they eventually develop cancer, follow a Mendelian inheritance pattern, with a 50% chance of transferring the genetic alteration to their offspring. However, it should be noted that *BRCA1* and *BRCA2* genes are found on autosomal chromosomes, and thus, mutations can be inherited from either the maternal or paternal side of a family [23, 30]. Other genes associated with higher cancer risk are *TP53, CHEK2, CDH1, ATM*, *PTEN*, and *PALB2* tumor suppressor genes [31, 32]. Lifetime cancer risks for BRCA1 and *BRCA2* mutation carriers in India have been studied in selected cohorts, showing elevated risks for breast and ovarian cancers compared to the general Indian population (Table 1).

**TABLE 1:** Estimated risk of cancer in Indian BRCA1*/2 m*utation carriers compared to the Indian general population.

Cancer type	Estimated lifetime risk in BRCA1 carriers (Indian data)	Estimated lifetime risk in *BRCA2* carriers (Indian data)	Estimated lifetime/cumulative risk in Indian general population (0–74 years)	References
Breast (female)	~45%–80% cumulative to age 80	~45%–70% cumulative to age 80	~2.9%	[33, 34]
Male breast	~1%–2% lifetime	~5%–10% lifetime	< 0.2%	[33, 35, 36]
Ovarian	~35%–45% cumulative	~15%–25% cumulative	~0.7% (GLOBOCAN India)	[33, 34]
Pancreatic	≤ 5% lifetime	~5%–10% lifetime	~0.97% (GLOBOCAN India)	[33, 37]
Prostate	Modest increase; cohort‐dependent	Markedly increased (~20%–40% lifetime in selected cohorts)	~0.67% (GLOBOCAN India)	[33, 34]
Melanoma	No consistent increased risk	Small increased risk (magnitude low)	~0.03% (extremely low in India)	[33, 38]

*Note:* BRCA1*/2* carrier risk for pancreatic cancer is derived from international data (Iqbal et al. [37]) as no large Indian cohort data are currently available.

While *BRCA* mutations are also linked to other malignancies, including male breast, pancreatic, and prostate cancers, the most clinically relevant impact in India is observed for female breast and ovarian cancers. This highlights the need for targeted genetic testing and management strategies within the Indian healthcare setting [39].

**FIGURE 2:**
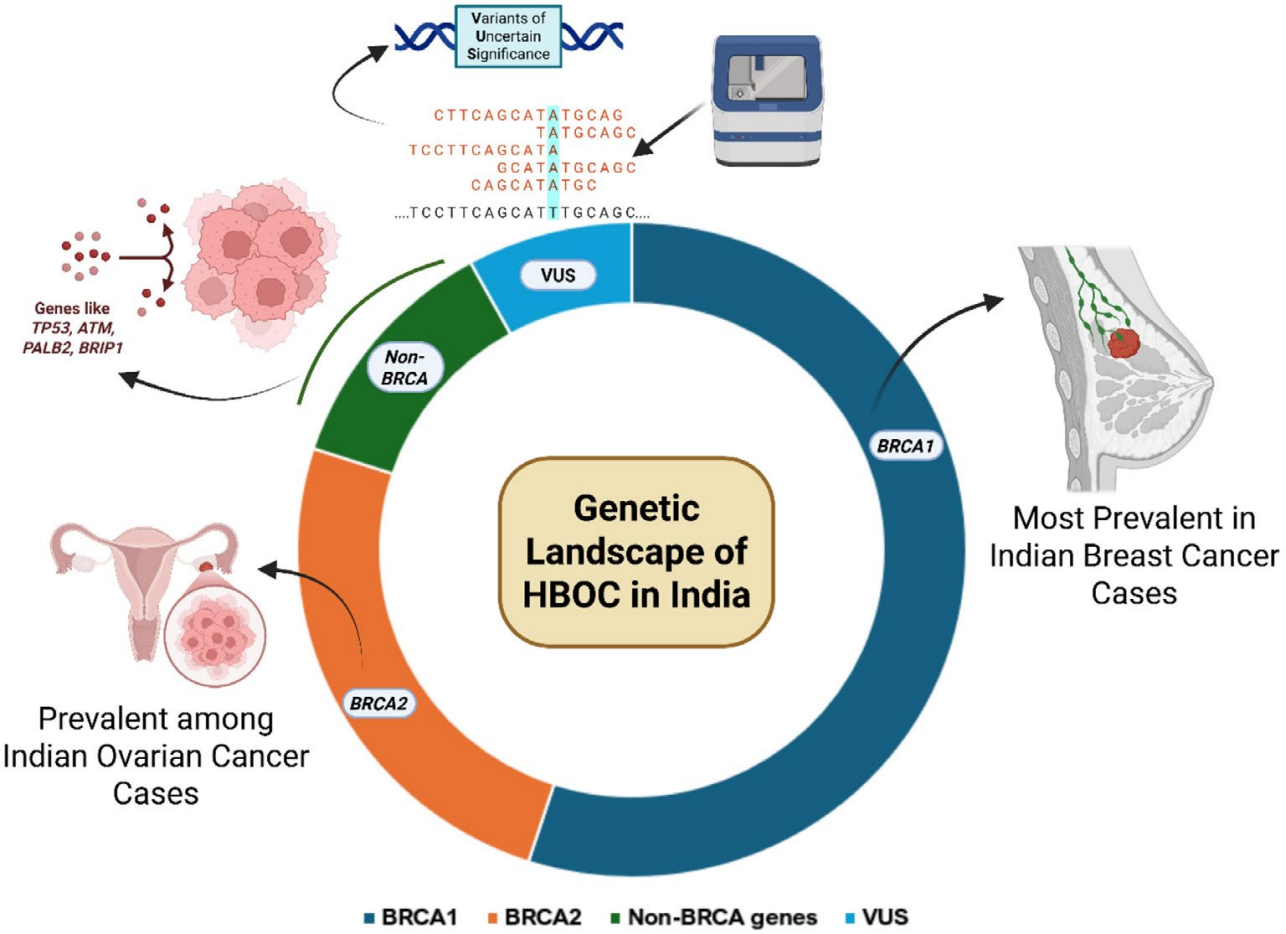
Genetic landscape of hereditary breast and ovarian cancer (HBOC) in India. This pie chart represents the estimated distribution of pathogenic genetic variants in Indian HBOC cohorts. *BRCA1* mutations constitute approximately 55% of all detected pathogenic variants, followed by *BRCA2* (25%), non‐*BRCA* genes including *TP53*, *PALB2*, *BRIP1*, and *ATM* (12%), and variants of uncertain significance (VUS) at 8%. Data are derived from recent Indian studies on HBOC cohorts [31, 43, 44, 45].

The prevalence of disease‐causing *BRCA1* or *BRCA2* mutations is estimated at 1 in 333–500 individuals in the general population, while among individuals of Ashkenazi Jewish ancestry it is approximately 1 in 40 [40]. Studies have shown that multigene panel testing can identify approximately 40%–50% more pathogenic variants compared to *BRCA1/2* testing alone, thereby improving diagnostic yield and clinical utility [15, 41, 42]. Additionally, the cancer mortality rate in India is largely due to late diagnosis and treatment delays. Thus, it's crucial to assess non‐*BRCA* genes alongside *BRCA* genes to comprehensively understand HBOC‐related mutations in the Indian population. The genetic landscape of HBOC in the Indian population compiled from recent studies is deciphered in Figure 2, which shows the significance and distributions of pathogenic variants in *BRCA1*, *BRCA2*, non‐*BRCA*, and Variants of Uncertain Significance (VUS) reflected across the reported studies. The proportions given in Figure 2 are compiled from several Indian cohort studies examining the genetic etiology of HBOC. *BRCA1* and *BRCA2* were more common, with two‐thirds of *BRCA*‐positive cases due to *BRCA1* mutations [43, 44, 45]. Non‐*BRCA* pathogenic mutations (such as *TP53*, *PALB2*, *ATM*, and *BRIP1*) occurred in 11.91% of cases in a non‐*BRCA* [46]. The percentage of VUS is estimated at a conservative 8% due to their common occurrence in multigene panel testing. All percentages were approximated to represent broad trends in the studies and to facilitate the development of a population‐appropriate genetic testing model for India. Incorporating gene panels covering both *BRCA1/2* and non‐*BRCA* genes can provide valuable insights into mutation spectra, aiding in patient management, risk assessment, and the identification of novel variants associated with HBOC. This approach facilitates better understanding and management of HBOC risk in both patients and their at‐risk family members [47].

The absolute lifetime cancer risk for HBOC varies depending on several factors, including the specific gene mutations involved, family history, and environmental influences. However, individuals with HBOC mutations, particularly in genes like BRCA1 and *BRCA2*, face significantly elevated risks compared to the general population. Estimates suggest that HBOC mutations provide a risk of 60%–80% for breast cancer and 20%–40% for ovarian cancer throughout the lifetime of affected individuals [35, 48]. Moreover, these pathogenic or likely pathogenic variants may also increase the risk of other cancers. Overall, HBOC pathogenic or likely pathogenic variants substantially heighten the lifetime cancer risk, underscoring the importance of early detection, genetic counseling, and preventive measures for individuals and families at risk [25, 30].

## 3 Clinical Management Strategies of HBOC

Management strategies for reducing breast cancer risk encompass three main processes: surveillance, chemoprevention, and finally, risk‐reducing surgery. Risk‐reducing mastectomy is particularly effective and can prevent over 90% of new breast cancer cases [49, 50]. In contrast, options for managing ovarian cancer (OC) risk are limited, with risk‐reduction salpingo‐oophorectomy being a viable option due to the absence of proven early diagnostic methods [49]. The majority of breast and ovarian cancers occur sporadically without any identifiable cause and often arise by chance. Consequently, genetic testing for BRCA1*/2* pathogenic or likely pathogenic variants will not be applicable to all individuals. It is primarily recommended for those with a history of HBOC. However, older women older than 60 years and diagnosed with triple‐negative breast cancer are advised to undergo genetic testing for *BRCA* mutations, despite their family history. Genetic testing helps identify inherited pathogenic or likely pathogenic variants in BRCA1*/2* or any cancer‐related genes. Genetic counselors help in determining the most appropriate type of genetic testing, interpreting test results, and guiding subsequent steps. If an inherited mutation is detected, it is advisable for the family members of the affected individual to undergo genetic testing to accurately assess their own risk levels. This initiative enables the better management of hereditary cancer risks within families [32, 49, 51].

### 3.1 Diagnostic Techniques

The discovery of pathogenic or likely pathogenic variants in the BRCA1 and *BRCA2* genes within families with several instances of early‐onset breast cancer marked a significant advancement in hereditary cancer genetics. For BRCA1*/2* mutation carriers, the cumulative risk of developing breast cancer by the age of 70 is estimated to be 60% and 55%, respectively, while for ovarian cancer, it is estimated to be 59% and 17%, respectively [48]. Genetic testing for BRCA1/2 pathogenic or likely pathogenic variants in females has crucial clinical implications. They should be offered options such as MRI and mammography, salpingo‐oophorectomy, and prophylactic mastectomy [52]. Moreover, *BRCA1/2* mutation carriers benefit from targeted PARP inhibitor‐assisted therapy [53]. However, *BRCA1/2* pathogenic or likely pathogenic variants contribute to only approximately 30% of high‐risk breast cancer families, with variation based on population demographics and selection criteria for patients predisposed to these cancers. The genetic testing criteria are depicted in Table 2. These findings highlight the complex landscape of hereditary cancer genetics and underscore the importance of comprehensive genetic testing and tailored treatment approaches in affected families [40, 53]. According to these studies, the frequency of *BRCA1/2*‐associated HBOCs is maximized in individuals with a personal or family history, for which specific criteria are used. Table 2 summarizes the criteria for genetic testing for HBOC. These criteria are informed by Indian clinical studies, case reports, and expert consensus recommendations, focusing on features most predictive of *BRCA1/2* pathogenic or likely pathogenic variants [23, 34, 42].

**TABLE 2:** Genetic testing criteria for HBOC syndrome.

No.	Criterion
1	Early‐onset breast cancer (< 45–50 years)
2	Triple‐negative breast cancer (< 60 years)
3	Male breast cancer at any age
4	Early‐onset ovarian cancer (any histology)
5	Multiple primary breast and/or ovarian cancers in an individual (e.g., bilateral breast cancer, breast + ovarian cancer)
6	Two or more first‐ or second‐degree relatives with breast and/or ovarian cancer
7	Known pathogenic BRCA1/2 mutation in the family

The diagnosis of pathogenic or likely pathogenic variants in the *BRCA1/2* genes, which are prevalent in most affected families, relies on the exploration of a heterozygous germline pathogenic variant in these genes through molecular genetic testing (Table 3). Individuals meeting established criteria, as previously mentioned, are considered ideal candidates for these tests. Testing typically begins with individuals exhibiting characteristics suggestive of HBOCs and, if feasible, extends to other family members. In cases where testing cannot be conducted on persons with a history of cancer, healthy family members may undergo testing [64].

**TABLE 3:** Studies on pathogenic BRCA1*/2* mutations in the Indian population.

Mutation/variant	Study region	Method employed	References	Key findings/notes
*BRCA1* c.68_69delAG, c.5266dupC; *BRCA2* c.5946delT, c.8537_8538delAG; 3 VUS identified	Across India	Targeted sequencing	[31]	29.1% BRCA mutation prevalence; common mutations in exon 10 (BRCA1) and exon 11 (BRCA2)
*BRCA2* c.5946delT; *ATM* c.7271T>G; other mutations in CHEK2, PALB2	Across India	Whole exome sequencing	[32]	Identified characteristic mutations specific to the Indian sub‐population
*BRCA1* c.68_69delAG, c.5137+1G>A; *BRCA2* c.5946delT; *PALB2* c.3113G>A; *ATM* c.7271T>G; *CHEK2* c.1100delC; 6 VUS identified	Across India	Amplicon sequencing	[45]	17.64% pathogenic mutations in non‐BRCA genes; identified population‐specific variants
*BRCA1* c.4158_4162delCTCTC, c.4327C>T (p.R1443X), c.1148_1149delAT (p.Asn383Argfs*6*), c.4399C>T (p.Gln1467X), c.4705_4706insTGGAATC (p.Ile1567fs5), c.5024_5025insT (p.Thr1675Thrfs4), c.68_69delAG (p.Glu23Valfs16), c.66_67delAG (p.Leu22Leufs*18), c.5118_5120delAAT; *BRCA2* c.6214_6218delCTTAA, c.5130_5133delTGTA, c.2621_2627delAACTGTC	Chennai, South India	PCR‐dHPLC	[54]	Spectrum of frameshift and nonsense mutations in both genes
*BRCA1* c.541G>T, c.1681delT, c.2295delG, c.4915C>T, exon 23 deletion; *BRCA2* c.932_933insT, c.9976A>T, c.10089A>G; 7 lethal mutations & 7 VUS	North India	PCR and DNA sequencing	[55]	Novel and recurrent mutations; no significant hormone receptor differences among carriers
*BRCA2* 950_951insA (Asn319fs), 1032_1033insA (Asn346fs)	North India	PCR and Sanger sequencing	[56]	Frameshift mutations detected in 6/41 samples
*BRCA1* 185delAG, 1014delGT, 3889delAG	North‐East India	PCR and DNA sequencing	[57]	Truncating mutations in exons 2 & 11; stop codons at amino acids 39, 303, 1265
*BRCA1* 180delA, 185delAG, 5370C>T; BRCA2 9097C>T	Malaysia (Indian ethnicity)	dHPLC and DNA sequencing	[58]	Ethnic Indian‐specific mutations observed
*BRCA1* c.68_69delAG, c.5266dupC, c.3683_3684dup; 11 VUS; *BRCA2* c.5946delT, 14 pathogenic variants; 4 VUS	Across Indian population	PCR/targeted sequencing	[59]	c.68_69delAG is a hotspot (12/51 cases); 31.87% deleterious *BRCA1/2* mutations; higher prevalence in cancer‐affected probands
*BRCA1/2* 56 novel mutations among 304 cases; 84.9% in *BRCA1/2*	Across India	Next‐generation sequencing	[60]	Majority of mutations in BRCA genes; includes novel variants
*BRCA1* c.68_69delAG; *BRCA2* c.5946delT; TP53 c.743G>A; 5 novel variants reported	Across India	Whole exome sequencing	[61]	Identified novel exonic variants contributing to HBOC in the West Indian population
*BRCA1* 185delAG	Malaysia (Indian ethnicity)	PCR + Sanger sequencing	[62]	Confirmed ethnic Indian‐specific variant
*BRCA1* c.5074+1G>A, c.2426A>G (p.Glu809Gly), c.68_69del, c.4224_4231del, c.4286A>G, c.1504_1508del, g.50962G>A (IVS14)	Hyderabad	PCR and Sanger sequencing	[63]	Multiple known pathogenic variants identified

Abbreviations: CSGE, conformation‐sensitive gel electrophoresis; dHPLC, denaturing high performance liquid chromatography; PCR, polymerase chain reaction.

Genetic tests are pivotal for identifying alterations in chromosomes or proteins. Molecular genetic tests, particularly in HBOC, analyze genes to pinpoint pathogenic or likely pathogenic variants causing diseases. In cases where no genetic alteration is detected, it is crucial to communicate to the patient that their risk of developing cancer remains comparable to that of the general population [65]. However, if a causal variant is identified, it becomes imperative to evaluate family members to ensure appropriate follow‐up care. Early detection of pathogenic variants is essential for implementing preventive measures, which can significantly impact outcomes for carriers. Therefore, genetic testing plays a pivotal role in guiding personalized management strategies and enhancing the overall care of individuals at risk for hereditary cancer syndromes [66].

Earlier studies have employed various diagnostic models to identify mutations responsible for HBOC within the Indian population, utilizing different testing methods, as outlined in Table 3. Previous results from sequencing 141 unrelated individuals and families with breast and ovarian cancer (BOC) using the TruSight Cancer panel, which includes 13 genes significantly associated with the risk of hereditary BOC [23]. Their study, employing multigene sequencing, identified pathogenic mutations in 51 (36.2%) patients, 19 of which were novel variants. The detection rate increased to 52% when only patients with familial breast cancer were included. Notably, detection rates were higher in younger age categories, with rates of 44.4% and 53.4% for patients aged under 40 and 40–50 years, respectively, compared to 26.9% for those aged over 45 years [23, 67].

In another study, the Illumina HiScan SQ system was used to screen 30 genes in 91 patients with hereditary cancers. Seventy‐four samples were evaluated using PCR‐dHPLC, revealing no deleterious mutations. In contrast, 17 samples were assessed for the first time, which identified 24 deleterious mutations, including 11 in BRCA1, four in *BRCA2*, and others in genes such as *TP53*, *RAD50*, *RAD52*, *ATM*, and *BRIP1*. Overall, 54.55% of the total patients who were previously evaluated and evaluated for the first time were found to have deleterious mutations, with the specific *BRCA1* mutation c.68_69delAG (p.Glu23Valfs*16) a frameshift variant, detected in approximately 22.73% of patients [60]. Moreover, whole‐exome sequencing (WES) of 30 patients with a family history of breast or ovarian cancer identified novel variants and genes associated with HBOC. Several highly oncogenic variants and genes linked to critical biological processes, such as DNA integrity, transcriptional regulation, the cell cycle, and apoptosis, were identified. Furthermore, a *BRCA*‐negative family with multiple breast cancer‐affected women was investigated, revealing a novel missense pathogenic variant in the *CHEK2*.

### 3.2 Chemoprevention and Hormonal Therapies

Various studies in the literature have shown that patients with hereditary breast cancer in India exhibit a significant prevalence of *BRCA1/2* mutations. The frequency of these mutations ranges from 2.9% to 28.0% among Indian familial breast cancer patients, with approximately 2.8% of early‐onset breast cancer patients in the Indian population showing *BRCA1/2* mutations. Notably, the incidence rate of *BRCA1* mutations appears to be greater than that of *BRCA2* mutations in Indian HBOC patients, possibly due to limited data availability on *BRCA2* mutations. Although there are slight variations in the population‐based *BRCA1/2* mutation spectrum, the overall prevalence of BRCA gene mutations remains consistent. This suggests that while there may be differences in the specific mutations identified, the overall occurrence of BRCA mutations is similar across different studies [59].

Earlier studies indicate that the local recurrence rate following breast‐conserving surgery in patients with breast cancer associated with *BRCA1/2* mutations is not notably greater than that in patients with sporadic breast cancer. Moreover, there was no discernible difference in prognosis between the various surgical methods [68, 69, 70, 71, 72, 73, 74, 75]. In HBOC patients, *BRCA1/2* mutations disrupt double‐strand DNA break repair mechanisms, and poly ADP‐ribose polymerase (PARP) plays a critical role in single‐strand DNA nick repair. The use of PARP inhibitors in HBOCs exploits this vulnerability by preventing DNA repair through synthetic lethality, ultimately leading to cell death [76]. PARPs, a family of 17 proteins, become activated in response to DNA damage. *BRCA1* and *BRCA2* are genes known for suppressing tumor growth, and they produce proteins essential for repairing DNA double‐strand breaks. Cells lacking functional *BRCA1* or *BRCA2* are vulnerable to inhibition of PARP activity. This vulnerability makes PARP inhibitors (PARPis) valuable for treating breast cancer patients who carry germline mutations in DNA repair genes, particularly those with harmful *BRCA1* and *BRCA2* mutations. These mutations are present in approximately 3%–4% of all women with breast cancer and account for 10%–20% of those with triple‐negative breast cancer [77]. Olaparib (brand name Lynparza) is an oral, small‐molecule inhibitor of PARP1/2. Initially, approved in 2014 for BRCA mutation‐positive ovarian cancer, Olaparib has shown efficacy in treating breast cancer patients with BRCA mutations [78].

Cancer chemoprevention refers to the use of medications to prevent or reduce the risk of cancer development [70, 71, 72, 73]. Tamoxifen, a generic drug, has been particularly effective in lowering the risk of breast cancer, especially in women identified as having a high risk of developing the disease [79]. Research indicates that taking tamoxifen for a period of 5 years can reduce the risk of breast cancer by up to 50% in high‐risk women [80, 81, 82, 83, 84]. For women with specific genetic mutations associated with breast cancer risk, such as *BRCA1* and *BRCA2* mutations, tamoxifen may offer additional benefits. Studies suggest that tamoxifen can help lower the risk of breast cancer in women with these mutations. However, the effectiveness of tamoxifen may vary depending on the specific mutation [35, 79, 80, 81, 84]. For instance, women with *BRCA1* mutations are more prone to developing hormone receptor‐negative cancers, which may reduce the effectiveness of tamoxifen in this subgroup [85]. In a phase III TNT trial comparing the efficacy of carboplatin and docetaxel in patients with triple‐negative breast cancer or those with metastatic recurrence and germline *BRCA1/2* mutations, significant improvements were noted in both the overall response rate (ORR) and progression‐free survival among those treated with carboplatin [47]. Specifically, the ORR was 68% in the carboplatin‐treated group and 33.3% in the docetaxel group, with a median PFS of 6.8 and 4.4 months, respectively. However, no disparity was observed in overall survival [47].

Inherited pathogenic variants linked to HBOC pose challenges for reproductive health, potentially necessitating interventions such as chemotherapy or oophorectomy. Women with these variants require counseling on cancer prevention, fertility preservation, and the risk of passing on the gene variants to offspring. Reproductive specialists guide patients on the timing of fertility preservation and preimplantation genetic testing, optimizing outcomes through tailored guidance. Gynecologic oncologists need to be aware of reproductive consequences, facilitating early referral to reproductive specialists for integrated treatment planning. Collaboration between oncologists and reproductive specialists ensures comprehensive care, addressing both cancer management and reproductive health needs. This interdisciplinary approach provides timely access to fertility preservation options and aids informed decision‐making. Educating patients about assisted reproductive technology empowers them to make informed choices, alleviate distress, and promote proactive management of reproductive health. Overall, holistic care considers medical and emotional aspects, aiming to optimize patient well‐being and quality of life [86].

### 3.3 Surveillance and Screening Recommendations

The recommended surveillance and screening approach for HBOC syndrome patients suggests initiating annual breast MRI or mammography (with tomosynthesis considered if MRI is unavailable) between the ages of 25 to 29 [34, 35, 87]. From ages 30 to 75, the recommendation is for annual mammography combined with breast MRI. To address the heightened risk of interval cancers, a proposal suggests alternating between MRI and mammographic screenings every 6 months, aiming for earlier detection [88]. Notably, this screening protocol begins a decade or more earlier than the ages recommended for average‐risk women by organizations such as the American Cancer Society and the European Reference Network (ERN) for Genetic Tumor Risk Syndromes (GENTURIS). While screening strategies vary based on geographical and institutional norms, a common practice involves staggering mammograms [88] and MRI scans at 6‐month intervals. Although some centres utilize screening ultrasound, its role as a primary screening modality remains uncertain [85]. The details are depicted in Table 4 based on the National Comprehensive NCCN Guidelines.

**TABLE 4:** HBOC surveillance and prevention strategies.

Gene	Breast cancer risk	Ovarian cancer risk	Other cancers	Recommendations for breast/ovarian cancer risk reduction
*BRCA1*	Elevated	Elevated	Prostate	Annual mammogram from 30 years or annual breast MRI from 25 years, Consider RRM Recommend RRSO within 35–40 years
*BRCA2*	Elevated	Elevated	Prostate, pancreas, melanoma	Annual mammogram from 30 years or annual breast MRI from 25 years, Consider RRM Recommend RRSO within 35–40 years (extend up to 40–45 years)
*ATM*	Elevated	Unknown/insufficient	Pancreatic, prostate	Annual mammogram from 30 years; consider breast MRI based on family history; risk‐reducing strategies individualized
*CDH1*	Elevated	Decreased	Diffuse gastric cancer	Annual mammogram and breast MRI from 30 years. Consider RRM (depending up on family history)
*CHEK2*	Elevated	Decreased	Colon cancer	Annual mammogram and breast MRI from 40 years. Consider RRM (based on family history)
*RAD51C*	Unknown	Elevated	N/A	Consider RRSO from 45 to 50 year
*RAD51D*	Unknown	Elevated	N/A	Consider RRSO from 45 to 50 years
*P53*	Elevated	Decreased	Adrenocortical carcinoma, leukemia, brain tumors, soft tissue sarcomas	Annual mammogram from 30 years and annual breast MRI from 20 to 29 years. Consider RRM
*PALB2*	Elevated	Unknown/insufficient	Unknown/insufficient	Annual mammogram/consider breast MRI from 30 years. Consider RRM (based on family history)
*STK11*	Elevated	Elevated (non‐epithelial)	Colorectal cancer	Annual mammogram/breast MRI starting at the age of 25 years
*MSH6*	Unknown/insufficient	Decreased	Colorectal cancer, endometrial cancer	Breast cancer management based on family history

Abbreviations: HBOC, hereditary breast and ovarian cancer; MRI, magnetic resonance imaging; N/A, not available; RRM, risk‐reducing mastectomy; RRSO, risk‐reducing salpingo‐oophorectomy.

## 4 Genetic Testing for HBOC

Genetic counseling plays a crucial role in assisting individuals and families affected by or at risk of genetic disorders, serving as a fundamental component of genomic medicine implementation. This process involves a comprehensive approach, incorporating the interpretation of family and medical histories to assess the likelihood of disease occurrence or recurrence. Furthermore, genetic counseling provides education on various aspects, including inheritance patterns, available testing options, management strategies, preventive measures, and available resources [89]. Additionally, counseling sessions aim to empower individuals to make informed decisions about their health, adapt to the implications of genetic contributions to disease, and provide support for reaching out to relatives who may also be at risk. Overall, genetic counseling serves as a vital tool in helping individuals understand and navigate the medical, psychological, and familial complexities associated with genetic disorders, ultimately promoting informed choices and enhancing support systems [89].

Genetic testing encompasses various approaches, including the study of physiological, immunological, or biochemical functions, as well as direct examination of an individual's genome [23]. These tests play a crucial role in detecting gene mutations that predispose individuals to cancer, identifying those who are at risk and in need of effective monitoring and risk management strategies. In the context of ovarian cancer, raising awareness among women and promoting regular screening can lead to early detection, significantly improving survival rates and enhancing quality of life. Therefore, genetic counseling and testing (GCT) have become essential, especially for patients with early‐onset disease or a family history of ovarian cancer. By delving into the genetic background of both the patient and their relatives, GCT provides valuable insights into the risk of developing the disease. Moreover, GCTs facilitate the expansion of collaborative care by involving healthcare providers in monitoring and surveillance programs. By identifying high‐risk individuals and implementing risk‐reduction measures, such as lifestyle modifications, GCTs empower patients and their families to take initiative steps in managing their health. This comprehensive approach not only aids in early detection and intervention but also ensures that patients receive the necessary support and resources to navigate their cancer journey effectively. Overall, integrating genetic counseling and testing into clinical practice for ovarian cancer patients enhances patient care and fosters an integrated approach to cancer management [22].

Studies in India have examined awareness and utilization of genetic counseling and testing (GCT) among at‐risk individuals for HBOC. Overall, knowledge of HBOC and BRCA testing remains limited, with a substantial proportion of eligible patients not accessing GCT services [88, 89]. Factors associated with higher awareness and uptake include younger age, higher educational level, and a strong family history of breast or ovarian cancer. Barriers identified include limited availability of trained genetic counselors, cost constraints, sociocultural beliefs, and apprehension regarding testing outcomes. Indian women with breast or ovarian cancer also report anxiety about genetic testing, uncertainty regarding its significance, and concerns about family implications, which influence their willingness to undergo counseling and testing [23, 31]. In addition to awareness and access, individual attitudes and perceptions towards genetic counseling and testing further influence uptake and engagement among at‐risk patients in India.

During genetic counseling for breast cancer patients, various attitudes towards GCTs were observed. Skepticism about the usefulness of genetic testing was common among those who declined immediately, while concerns about the potential impact on their future led others to hesitate, preferring to postpone testing. Some participants, referred to as late decliners, withdrew from the study due to fear of test results. Identifying these individuals proved challenging, as their behavior resembled that of patients who had already received results [88]. However, most participants agreed with the timing of counseling or expressed a preference for earlier access to genetic counseling. These findings underscore the complex interplay of attitudes, perceptions, and concerns surrounding GCT among cancer patients, highlighting the importance of tailored approaches to counseling and testing to address individual needs and preferences [88, 89].

### 4.1 Guidelines/Testing Criteria for Patients With Breast and Ovarian Cancer

The NCCN Guidelines represent a comprehensive framework detailing sequential management decisions and interventions applicable to 97% of cancer‐affected patients in the United States. These guidelines are continuously updated to reflect the latest advancements in cancer research and management, ensuring relevance and accuracy. They cover a wide range of aspects, including cancer screening, prevention, supportive care, and specific populations, and offer guidance to various stakeholders involved in decision‐making, such as physicians, nurses, pharmacists, payers, patients, and their families. Additionally, specific NCCN Guidelines have been tailored for HBOC syndrome genetic testing in India and other nations, exemplifying their adaptability and relevance on a global scale [22].

### 4.2 Importance of Genetic Testing and Counseling

Genetic counseling plays a pivotal role in enhancing patient–doctor communication by bridging gaps often present in traditional medical consultations alone. While medical consultations focus on treatment options, they may not adequately address the underlying causes and complexities of genetic diseases. Through genetic counseling, patients gain a deeper understanding of their personal and family health history, empowering them to make informed decisions about their healthcare. By leveraging this information, doctors can refer patients to genetic testing when necessary, enabling the identification of hereditary conditions and facilitating accurate diagnoses. This ensures that patients receive appropriate treatment tailored to their genetic makeup, ultimately improving their chances of obtaining effective medical care [23, 90, 91].

## 5 Recent Advances in Genetic Testing for HBOCs

### 5.1 Next‐Generation Sequencing (NGS) Technologies

Next‐generation sequencing (NGS) has emerged as a vital tool in medical genetics, enabling comprehensive analysis of multiple genes and sequencing of entire exomes (WES), transcriptomes, or genomes (WGS). This high‐throughput approach is particularly valuable for revealing the genetic underpinnings of complex, multigene diseases such as cancer. Of particular interest are tumors with a familial predisposition, as they offer opportunities to identify novel genes or gene variants implicated in cancer pathogenesis at the germline level [92]. Discovering these genetic factors holds significant translational value, aiding clinicians in refining risk assessment and prevention strategies. Despite the identification of highly penetrative genes such as BRCA1 and BRCA2 in some familial breast and ovarian cancer cases, a considerable proportion of cases, termed non‐BRCA cases, remain unexplained. However, through NGS technologies such as gene panels, WES, and WGS, researchers can explore these non‐BRCA patients, potentially revealing novel genetic factors contributing to familial breast and ovarian cancer [92, 93, 94]. Several studies have revealed novel susceptibility genes associated with HBOC, such as XRCC2, FANCC, and BLM [95, 96, 97, 98, 99, 100]. Several representative studies employing NGS technologies in HBOC are summarized in Table 5. Despite the promise of WES and WGS as comprehensive genetic diagnostic tools, their widespread implementation faces challenges including high costs, the complexity of bioinformatics pipelines, and large data‐storage requirements.

**TABLE 5:** List of NGS studies in hereditary breast and ovarian cancer.

Mutation	Associated with HBOC/key findings	Study region	Method employed	References
*BRCA1* c.68_69delAG, c.5266dupC; *BRCA2* c.5946delT, c.8537_8538delAG; 3 VUS	29.1% BRCA mutation prevalence; common mutations in exon 10 (BRCA1) and exon 11 (BRCA2)	India	Targeted sequencing	[31]
*BRCA1/2* (unspecified variants)	High prevalence of BRCA mutations among ovarian cancer patients (25.2%) irrespective of family history	Pune, India	Multicentric cross‐sectional study	[48]
*BRCA1* c.68_69delAG, c.5137+1G>A; *BRCA2* c.5946delT; PALB2 c.3113G>A; *ATM* c.7271T>G; *CHEK2* c.1100delC; 6 VUS	17.64% pathogenic mutations in non‐BRCA genes; population‐specific variants	India	Amplicon sequencing	[52]
*BRCA1* c.68_69delAG, c.5266dupC; *BRCA2* c.5946delT; 4 VUS	31.87% deleterious BRCA1/2 mutations; higher prevalence in cancer‐affected probands	India	Targeted sequencing	[50]
*BRCA1* c.68_69delAG; *BRCA2* c.5946delT; *TP53* c.743G>A; 5 novel variants	Novel exonic variants contributing to HBOC in West Indian population	India	Whole exome sequencing	[63]
*MRE11A* (c.1090C>T; p.Arg364Ter)	Truncated protein lacking DNA‐binding domains; loss of function	India	NGS	[101]
*BRCA1* c.68_69delAG, c.5266dupC, c.181T>G; *BRCA2* c.5946delT and other recurrent pathogenic variants	Comprehensive mutation spectrum analysis of BRCA1/2 in Indian HBOC patients. Identified recurrent pathogenic variants and highlighted population‐specific mutation patterns relevant for hereditary breast and ovarian cancer screening and risk assessment	India	NGS‐based BRCA1/2 mutation analysis	[102]
*BRCA1/2* (unspecified variants)	23.8% prevalence of BRCA mutations; significant correlation with family history	South India	Retrospective cohort study	[103]
*BRCA1/2* (unspecified variants)	16.6% prevalence of pathogenic BRCA1/2 mutations in unselected breast cancer cohort	India	Unselected cohort study	[104]
*BRCA1/2* (unspecified variants)	BRCA mutation prevalence ranging from 10% to 18%; highlights need for broader genetic testing	India	Population‐based review	[105]

### 5.2 Multigene Panel Testing

Multiple gene panels for HBOC testing involve the simultaneous analysis of multiple genes associated with a hereditary predisposition to breast and ovarian cancers. These panels typically include genes beyond the well‐known *BRCA1/2* and *BRCA2* genes, such as *ATM, CHEK2, PALB2, TP53*, and others, which are known to confer varying levels of cancer risk when mutated [106]. By analyzing multiple genes simultaneously, multigene panels offer a comprehensive approach to genetic testing, allowing for the identification of mutations in genes beyond BRCA1/2 that may contribute to hereditary cancer risk. This approach enhances the diagnostic yield, enabling healthcare providers to offer more personalized risk assessment and management strategies for individuals with a family history of HBOC. Additionally, multigene panels help uncover genetic factors that may have been overlooked with single‐gene testing, providing valuable insights into the genetic basis of HBOCs [107, 108, 109, 110].

### 5.3 Liquid Biopsy and Circulating Tumor DNA Analysis

**FIGURE 3:**
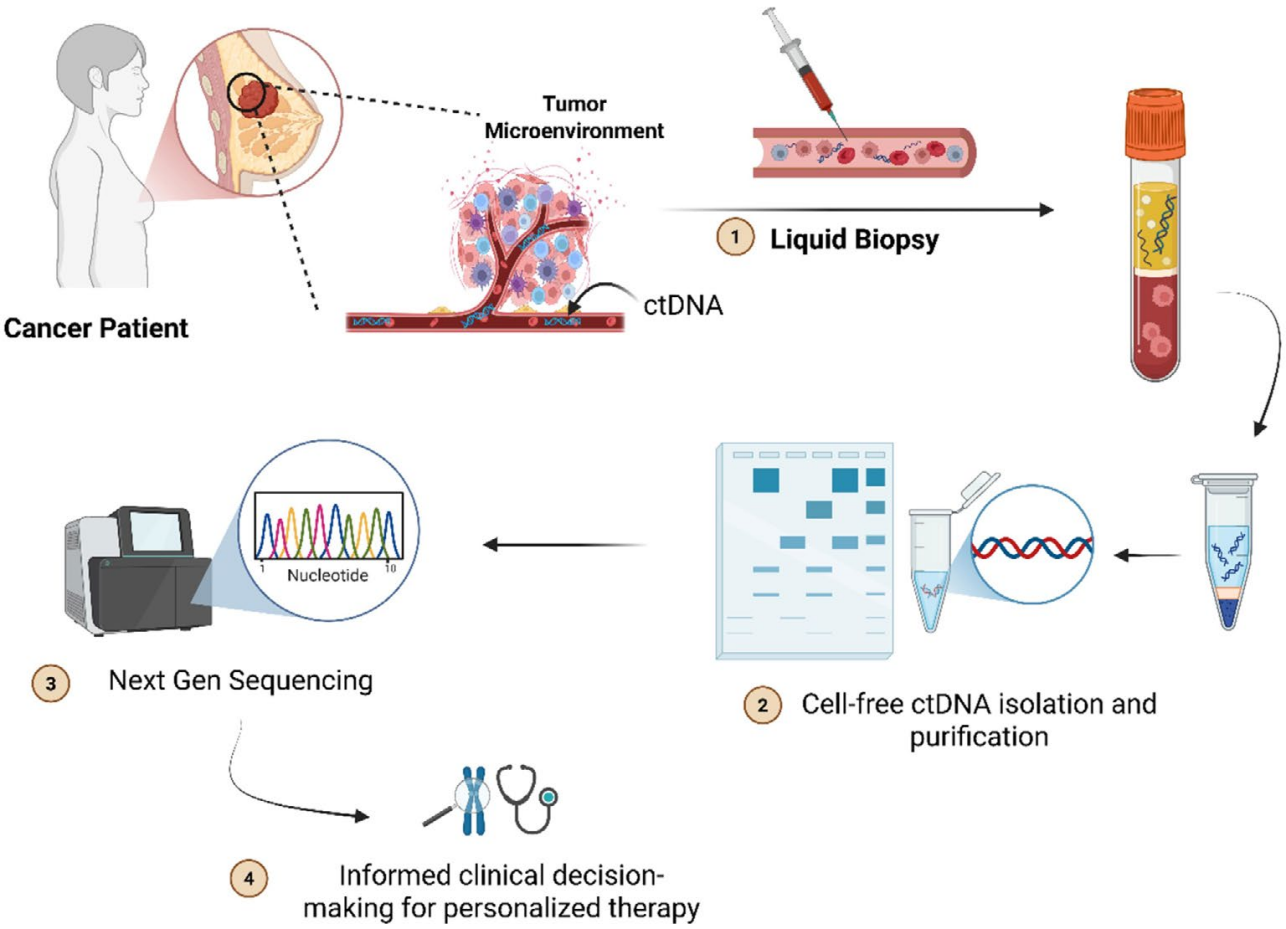
Workflow of liquid biopsy and ctDNA analysis in HBOC management. Tumor cells release circulating tumor DNA (ctDNA) into the bloodstream. This DNA is extracted through liquid biopsy using blood samples (Step 1), followed by cell‐free DNA isolation and purification (Step 2). The extracted ctDNA is sequenced using next‐generation sequencing (NGS) platforms (Step 3), enabling the detection of actionable mutations. This genomic data informs personalized clinical decision‐making, including surveillance strategies and targeted therapies (Step 4). Scheme is adapted from Ref. [114].

Tissue biopsy serves as the conventional method for diagnosing cancers and provides material for genotyping, aiding in targeted cancer therapies. However, its limitations lie in assessing cancer development, prognosis, and genotyping due to tumor heterogeneity and evolution. Circulating tumor DNA (ctDNA) refers to single‐ or double‐stranded DNA released by tumor cells into the bloodstream that carries mutations from the original tumor [111]. Liquid biopsy based on ctDNA analysis has emerged as a promising approach for molecular cancer diagnosis and monitoring. Studies have demonstrated that screening for genetic mutations using ctDNA is highly sensitive and specific, suggesting its potential to enhance current tumor diagnostic systems, including early‐stage detection. Furthermore, ctDNA analysis accurately tracks tumor progression and prognostic outcomes and can guide targeted therapy decisions. Thus, leveraging ctDNA as a liquid biopsy could revolutionize tumor management [112, 113]. The potential of utilizing ctDNA as a predictive and prognostic marker for monitoring breast cancer, as well as for predicting cancer recurrence and metastasis, was also examined. This could emerge as a pivotal component of a precision‐oriented approach to HBOC treatment [100]. The flow for liquid biopsy, ctDNA analysis, and genetic testing is portrayed in Figure 3.

## 6 Recent Advances in the Clinical Management of HBOC

In response to the treatment challenges faced by individuals with HBOCs in India, expanding access to genetic testing and genetic counseling services has emerged as a viable solution. Genetic testing facilitates the early detection of cancer risk within affected families, allowing healthcare providers to offer tailored treatment options and counseling to patients. Early detection is crucial for reducing mortality rates and enhancing treatment outcomes, a goal that can be achieved through the implementation of a comprehensive screening program. Additionally, raising awareness about inherited breast and ovarian cancers among the Indian public and medical community is imperative and can be achieved through educational campaigns, seminars, and workshops [23].

The integration of genetic testing into standard cancer care protocols has the potential to enhance diagnostic precision and enable personalized treatment plans for individuals with HBOC. Collaborative efforts between research institutions and healthcare providers can further drive the development of customized treatment approaches tailored to the Indian context. Overcoming the financial barriers associated with genetic testing and various treatment options is essential. The accessibility and affordability of genetic testing and targeted therapeutics can be enhanced through policy modifications and partnerships with pharmaceutical companies. Establishing specialized centres and programs dedicated to HBOC care, offering comprehensive genetic screening, counseling, and healthcare benefits, will be instrumental. These centres can also maintain a central patient registry to track genetic abnormalities and deliver personalized therapy, contributing to ongoing research and clinical trials. By implementing these measures, the understanding and management of HBOC in India can be significantly improved, leading to better treatment outcomes and higher survival rates [115].

HBOC pose a significant health risk, and personalized medicine approaches offer tailored strategies for its management. Through genetic testing, individuals with a family history of HBOC can undergo screening for mutations in genes such as BRCA1/2, allowing for early detection and intervention. Once identified, patients can benefit from personalized risk assessment, which considers genetic, lifestyle, and environmental factors to create a targeted surveillance and prevention plan. For those testing positive for mutations, personalized treatment options such as prophylactic surgery or enhanced screening protocols can mitigate risk. Furthermore, advancements in pharmacogenomics have enabled the development of tailored therapeutic interventions, such as PARP inhibitors, which exploit genetic vulnerabilities specific to HBOCs, improving treatment efficacy and patient outcomes. Integrating these personalized medicine approaches not only enhances risk assessment and management but also empowers individuals with HBOCs to make informed decisions regarding their health and well‐being [116].

The importance of providing precise healthcare to patients with HBOCs along with their families has been widely acknowledged at both the national and international levels. The Centers for Disease Control and Prevention (CDC) office of public health genomics has categorized HBOCs as Tier 1 genomic applications, recognizing the value of early detection and tailored public health interventions [117]. While there have been notable advancements in care, particularly in identifying actionable genetic variants in the germ line, there are still significant gaps in knowledge regarding the continuum of care for these syndromes. These gaps include identifying individuals at risk of carrying familial pathogenic variants, assessing risks for primary and subsequent cancers, exploring medical and lifestyle interventions to mitigate cancer risks, addressing the psychosocial needs of patients and families, and ensuring that underserved populations with these syndromes receive adequate care [118].

### 6.1 Management of HBOC in India: Current State, Limitations, and Opportunities

Currently, HBOC syndrome, primarily associated with BRCA1 and BRCA2 mutations, represents a significant public health concern in India. Recent studies indicate a higher prevalence of BRCA mutations in Indian populations compared to Western countries. For instance, one study reported that 59.3% of mutations were in BRCA1, and 40.6% in BRCA2, with missense mutations being the most common in both genes [34, 54, 55]. Despite this, the implementation of genetic testing and counseling services remains limited. A consensus statement from the Indian Society of Medical and Pediatric Oncology highlighted several barriers, including insufficient infrastructure, financial constraints, and a lack of trained genetic counselors. These challenges hinder the widespread adoption of genetic testing and personalized management strategies for HBOC patients in India [23, 119]. The limitations in managing HBOC in the current Indian scenario are that there is a clear lack of access to genetic testing and counseling. In India, the number of counselors and urban‐centric services is scarce, combined with high testing costs [31, 61]. Additionally, there is limited awareness of hereditary risks among patients and healthcare providers, as well as a lack of standardized national guidelines for HBOC testing and management [34, 42, 59]. To overcome these challenges, there should be an expansion of genetic counseling training in medical curricula and professional development. Along with this, public awareness campaigns promoting hereditary cancer risk awareness and preventive testing must be promoted, aiming for the development of national guidelines and policies to standardize care. Moreover, to overcome the research gaps, more data should be generated on BRCA and non‐BRCA mutations in diverse Indian populations to inform screening and therapy. By addressing these limitations and leveraging the opportunities, we can improve the status of early detection, personalized management, and overall outcomes for HBOC patients and their families in India.

## 7 Conclusion and Future Directions

**FIGURE 4:**
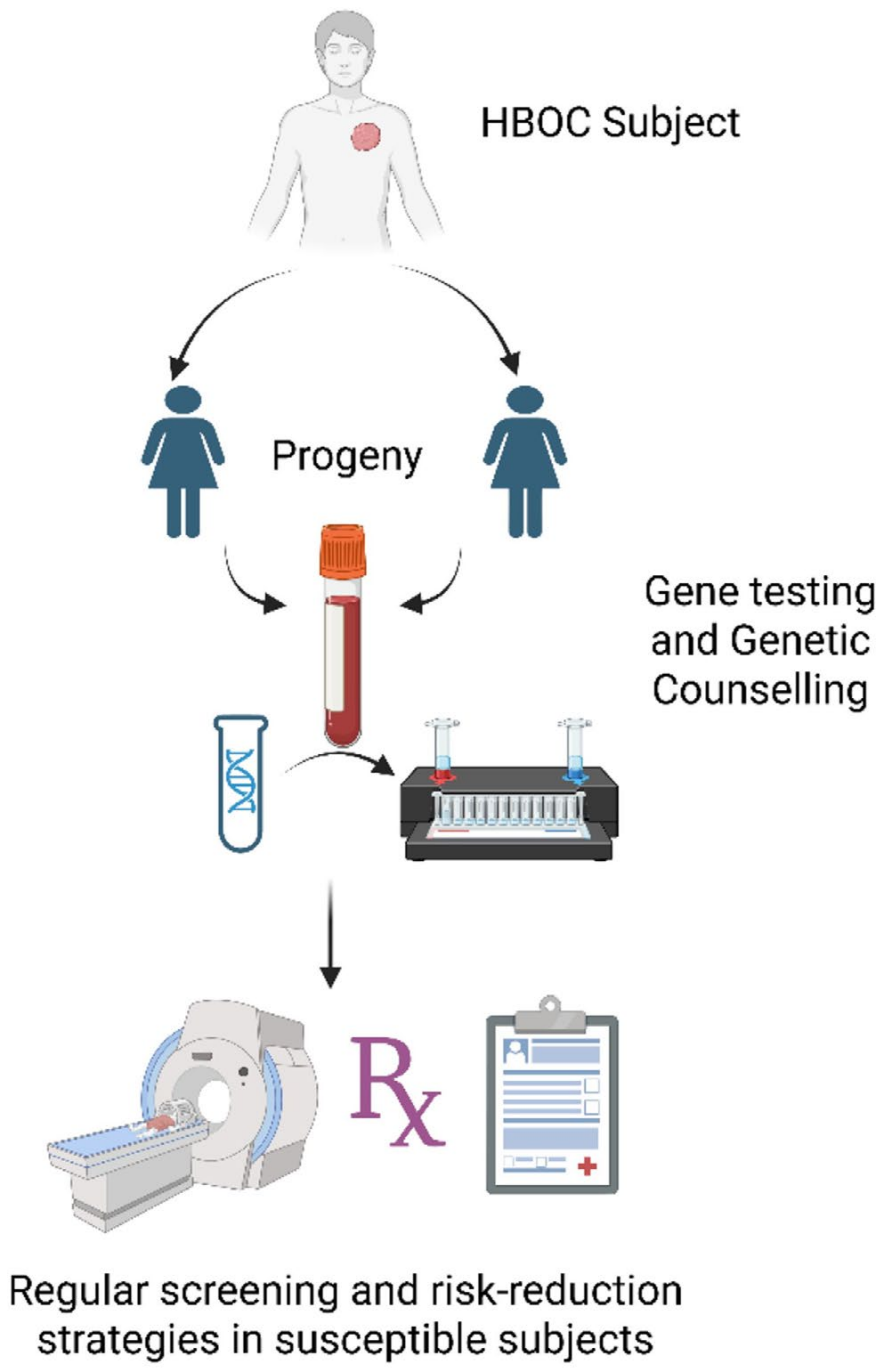
Family‐based genetic screening and future directions in HBOC care. This schematic illustrates the cascade testing and surveillance strategy essential for improving hereditary breast and ovarian cancer (HBOC) outcomes in India. Once a pathogenic mutation is identified in an index HBOC patient, first‐degree relatives (progeny) undergo genetic testing and counseling. Mutation‐positive individuals are offered targeted surveillance (e.g., frequent screening, mammography, scans, imaging, etc.) and risk‐reduction strategies (e.g., chemoprevention or prophylactic surgery). Future efforts should focus on strengthening infrastructure for family‐based testing, improving access to genetic services, and implementing population‐specific preventive strategies to enable timely interventions in high‐risk individuals.

Recent advancements in genetic testing and clinical management of HBOC in India have provided significant insights into the prevalence and molecular underpinnings of these malignancies. Studies have highlighted the substantial burden of BRCA1/2 mutations among Indian patients with hereditary breast cancer. Additionally, the emergence of genetic testing technologies has facilitated the identification of germline mutations, aiding in risk assessment, early detection, and personalized treatment strategies (Figure 4). The integration of PARP inhibitors, such as Olaparib, has revolutionized the therapeutic landscape for individuals with BRCA mutations, offering targeted and effective interventions that capitalize on the vulnerabilities of DNA repair pathways. Continued efforts in genetic research, coupled with enhanced access to genetic counseling and testing services, hold promise for advancing the clinical management of HBOC in India. Collaborative initiatives aimed at raising awareness, improving screening protocols, and implementing risk‐reducing strategies will be crucial in reducing the burden of HBOCs. Moreover, the development of tailored approaches that consider the unique genetic and environmental factors influencing disease risk and progression will be essential for optimizing patient outcomes. By leveraging the latest insights from genetic testing and integrating them into comprehensive clinical management protocols, healthcare professionals can strive towards more effective prevention, early detection, and personalized treatment of HBOC, ultimately improving the quality of life for affected individuals and their families [23, 120, 121].

Future directions in the realm of recent advances in genetic testing and clinical management of HBOC in India hold significant promise for advancing patient care and outcomes. One key avenue of exploration involves further elucidating the genetic landscape of HBOC in the Indian population, with a focus on identifying novel genetic variants and understanding their implications for disease risk and management. Collaborative research efforts, leveraging advanced genomic technologies and large‐scale population studies, will be instrumental in this endeavor, enabling the development of more targeted and personalized approaches to screening, prevention, and treatment. Moreover, efforts to enhance access to genetic counseling and testing services, particularly in underserved regions, will be crucial for ensuring that individuals at risk of HBOC receive timely and comprehensive care. This includes leveraging telemedicine and digital health platforms to reach remote populations and implementing culturally sensitive and linguistically appropriate interventions. Additionally, initiatives aimed at raising awareness about hereditary cancer syndromes, empowering patients and healthcare providers with knowledge and resources, and fostering multidisciplinary collaborations between oncologists, geneticists, and reproductive specialists will be essential for optimizing the management of HBOCs and improving patient outcomes across India.

As genetic information increasingly integrates into clinical practice in the context of HBOCs, paramount attention must be given to privacy and ethical considerations. Safeguarding patient confidentiality, ensuring informed consent, and protecting against potential misuse or discrimination based on genetic data are imperative. Future research directions should focus on refining genetic testing technologies, expanding access to and testing services, and elucidating the impact of genetic variants on disease risk and treatment outcomes. Policy development should aim to establish clear guidelines for the responsible use of genetic information, promote equitable access to genetic services, and address legal and regulatory challenges surrounding data privacy and informed consent. Collaborative efforts among policymakers, researchers, healthcare providers, and patient advocacy groups are crucial for navigating these complex issues and maximizing the benefits of genetic information while upholding ethical principles and safeguarding patient rights in the management of HBOCs.

## Author Contributions

**Prathika Sherigar:** conceptualization, writing – original draft, writing – review and edit, formal analysis. **Srikrishna Kedlaya Herga:** writing – review and edit, visualization, formal analysis. **Karthik S. Udupa:** conceptualization, writing – original draft, writing – review and edit, supervision, formal analysis. **Ananth Pai** and **Sharada Mailankody:** writing – review and edit, formal analysis.

## Funding

The authors have nothing to report.

## Conflicts of Interest

The authors declare no conflicts of interest.

## Data Availability

The authors have nothing to report.
